# Case report: Surgical closure of a facial defect in an African wild dog (*Lycaon pictus*) utilizing a transpositional skin flap

**DOI:** 10.3389/fvets.2024.1470528

**Published:** 2025-01-07

**Authors:** Armen M. Brus, Maureen Spinner, Tess Rooney, Kimberly A. Thompson

**Affiliations:** ^1^College of Veterinary Medicine, Michigan State University, East Lansing, MI, United States; ^2^School of Veterinary Medicine and Biomedical Sciences, Texas A&M University, College Station, TX, United States; ^3^Binder Park Zoo, Battle Creek, MI, United States; ^4^San Diego Zoo Wildlife Alliance, San Diego, CA, United States; ^5^School of Veterinary Medicine, University of California, Davis, Davis, CA, United States

**Keywords:** *Lycaon pictus*, transpositional flap, wound, wound management, secondary closure

## Abstract

Veterinary intervention in zoological species can be complicated by species-specific social dynamics. African wild dogs are a pack species and removal or separation of an individual may disrupt established pack hierarchy resulting in conspecific aggression. Therefore, medical interventions that optimize a quick return to health are ideal to minimize the duration of absence from the pack. These principles were utilized for a three-year-old male intact zoo-housed African wild dog (AWD) that presented with an episode of severe, acute, right-sided facial swelling. Swelling initially responded to medical management, however 6 days later the facial swelling worsened. The AWD was anesthetized and required extensive debridement of necrotic tissue. The absence of deep bacteria on histopathologic evaluation and a negative bacterial culture was suggestive of envenomation. The resultant open wound was managed every other day with debridement and topical manuka honey covered with a tie-over bandage. Each wound therapy treatment required general anesthesia but resulted in a healthy granulation tissue bed 9 days following initial debridement. Rather than proceeding with second intention healing and continued bandage changes, a transpositional skin flap surgical procedure was performed and enabled full closure of the large skin defect with complete tissue apposition and a good cosmetic outcome. The surgery reduced the number of anesthetic events and time that would have been required for second intention healing of the defect, which enabled a more rapid and ultimately successful reintegration of this individual into the pack without any perceived changes to the hierarchical structure.

## Introduction

African wild dogs are an endangered wild canid species native to sub-Saharan Africa that live in large familial packs containing as many as 30 individuals. This highly social species is well-known for its established pack hierarchies (1, 2). Because of the strong social bonds in AWDs, veterinary care is managed by balancing the goal of optimized and expedited healing and welfare for an individual, while minimizing the number of anesthetic events and overall time that an individual is separated from their pack. Some evidence suggests that visual coat recognition of individual AWD pack members is important to maintaining social hierarchies (1). Therefore, maintenance of cosmesis during reconstructive procedures may be an additional consideration in this species. In zoological facilities, AWDs have been reported to require treatment of wounds from conspecific aggression, insect bites, envenomation, or trauma inflicted by plants or other foreign bodies, such as grass awns (2, 3). Examination and treatment of wounds in AWDs can sometimes be managed medically, but substantial wounds typically require general anesthesia (2, 3). Disruptions in social hierarchies can occur during separation of individuals, resulting in social structure changes and trauma from conspecific aggression, which may ultimately end in an individual failing to be accepted back into the pack (1).

Facial wounds amongst domestic dogs are a common injury complicated by the limits of the soft tissue of the head and the close association to structures including the mouth, nose, ears, and eyes (4). A variety of surgical techniques are used to aid in the management and closure of facial skin wounds and defects in domestic dogs including axial pattern flaps, subdermal plexus flaps, and skin grafts (4–6). Reduced healing time compared to the prolonged time needed for second intention healing of larger wounds may make surgical repair methods more desirable in a zoological setting (5–9).

Local subdermal plexus flaps used to repair facial wounds in domestic dogs, including transpositional, advancement, and rotational flaps, have all largely resulted in appropriate cosmesis and healing (6, 8, 9). Transpositional skin flaps require creation of a rectangular skin flap that is rotated within 90 degrees into an adjacent defect around a pivot point. Advancement flaps, more simply, are created and advanced parallel to a wound without rotation or need for secondary donor site closure. Combining the principles of these previous techniques results in creation of a rotational flap utilizing a semicircular incision, which enables both advancement and rotation of tissue into an adjacent defect without the need for secondary donor site closure (6).

This report describes the use of a transpositional skin flap for closure of a large facial defect on an AWD. This procedure resulted in excellent cosmesis while simultaneously reducing the number of anesthetic events, time to achieve healing, and duration of separation away from the pack. Reducing these variables allowed for the AWD to be successfully re-introduced to the pack sooner and likely helped to minimize shifts in the hierarchical order of the pack.

## Case presentation

A three-year-old, male intact zoo-housed AWD presented with an acute onset of severe, right sided facial swelling, pawing at the face, and emesis. Based on the acute clinical presentation, no obvious wounds and low probability for fungal disease based on geographic location, treatment for a suspected allergic hypersensitivity reaction was initiated and included: prednisone [0.5 mg/kg *per os* (PO) twice daily; Jubilant Cadista Pharma], diphenhydramine (2 mg/kg PO twice daily, Major Pharmaceuticals), and maropitant citrate (Cerenia; 2 mg/kg PO once daily; Zoetis) for 3 days. Emesis resolved within hours and facial swelling decreased by approximately 75% over the following 72 h.

**Figure 1:**
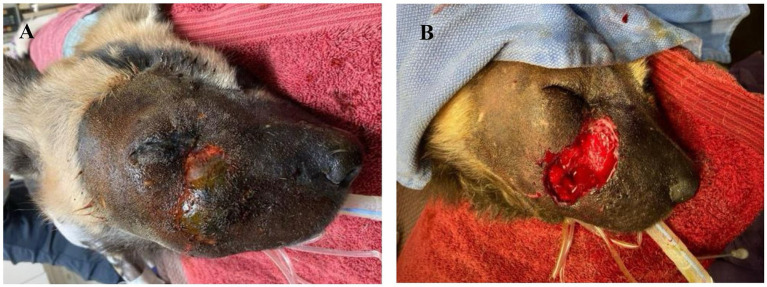
**(A)** Initial anesthetized evaluation of facial swelling in an African wild dog 6 days after attempted medical management. **(B)** Immediately post-debridement of necrotic and devitalized tissues.

Six days after initial presentation, the facial swelling worsened in severity and the AWD became lethargic and anorexic. The dog was anesthetized for examination via induction dart containing ketamine 2 mg/kg (ZooPharm), dexmedetomidine 4 mcg/kg (Dexdomitor, Zoetis), midazolam 0.14 mg/kg (ZooPharm), butorphanol 0.2 mg/kg (Vetorphic, VetOne) intramuscularly (IM) and intubated, and anesthesia was maintained with isoflurane (11). Baseline diagnostics were obtained. Complete blood count revealed a marked leukocytosis (26 k/μL; reference range, 5.0–18.1 k/μL) characterized by a mature neutrophilia (23 k/μL; reference range, 2.6–7.5 k/μL) and monocytosis (1,584/μL; reference range, 0.2–0.5 k/μL) and a mild elevation in fibrinogen (321 mg/dL; reference range, 110–316 mg/dL). Serum chemistry, coagulation panel, and urinalysis were unremarkable except for elevated D-dimers (486 ng/mL; reference range <250 ng/mL) (2). Dental examination and thoracic and skull radiographs were unremarkable except for soft tissue swelling over the right dorsolateral nose and muzzle. The dog was assessed to be mildly dehydrated and hyperthermic (rectal temperature 103°F). On physical examination there was diffuse, firm facial swelling and pitting edema that encompassed the entire right oral labia to the dorsal aspect of the muzzle and extended to the dorsocaudal right periocular region (Figure 1A). A central 4 × 6 cm area had devitalized skin with a firm, dry texture and green to black discoloration, which contained thick, purulent, red to white exudate upon aspiration. The area was clipped and prepared aseptically with dilute betadine and surgical debridement of all devitalized tissue was performed (Figure 1B). The excised skin and tissue were placed in 10% buffered formalin for histopathologic evaluation. Aerobic and anaerobic cultures of the deep tissues of the incision were obtained. A four cm subdermal pocket was present and lavaged copiously with sterile saline. The pocket was packed with antimicrobial medical tape (Covidien, Curity AMD Antimicrobial Packing Strips 0.2% Polyhexamethylene Biguanide HCl). The entire wound was covered with a colloid manuka honey primary layer (Medi-honey hydrogel colloid sheet; Derma Science Inc.), and sterile gauze was used to create a tie-over bandage with nylon (Ethilon polyamide6; Eticon) suture loops and umbilical tape (Jorvet). Treatments included: fluids (Lactated Ringer’s Solution 16 mL/kg IV once, 36 mL/kg SQ every 48 h for 3 doses; Novatech), ceftiofur crystalline free acid (9.7 mg/kg SQ once; Zoetis), meloxicam (0.1 mg/kg SQ every 48 h; Covetrus), famotidine (0.8 mg/kg SQ every 48 h for 3 doses; Westward), and maropitant citrate (Cerenia; 1 mg/kg SQ every 48 h for 3 doses; Zoetis).

The patient was administered atipamezole (0.04 mg/kg IV; Antisedan, Zoetis) and recovered normally. Due to the necessity for future frequent repeated anesthetic procedures, the dog was housed in isolation from the rest of the pack at the zoo’s veterinary hospital. The AWD was anesthetized as previously described every 48 h for parenteral medication administration due to continued anorexia, and for wound care including assessment, debridement, and tie-over bandage changes. The AWD’s anorexia resolved after 8 days of treatments and the wound continued to show subjective improvement and healing.

The histopathology report revealed changes consistent with severe necrotizing dermatitis with chronic inflammation. The aerobic and anaerobic cultures resulted in no growth and PAS stain was negative for fungal organisms. Gram stain revealed a few gram-positive bacteria only within the superficial ulcerated tissue. Dermatopathological evaluation concluded that the presence of necrotizing dermatitis was non-specific, but the lack of organisms within the deeper tissues considered concurrently with the clinical presentation supported possible envenomation associated tissue injury.

**Figure 2:**
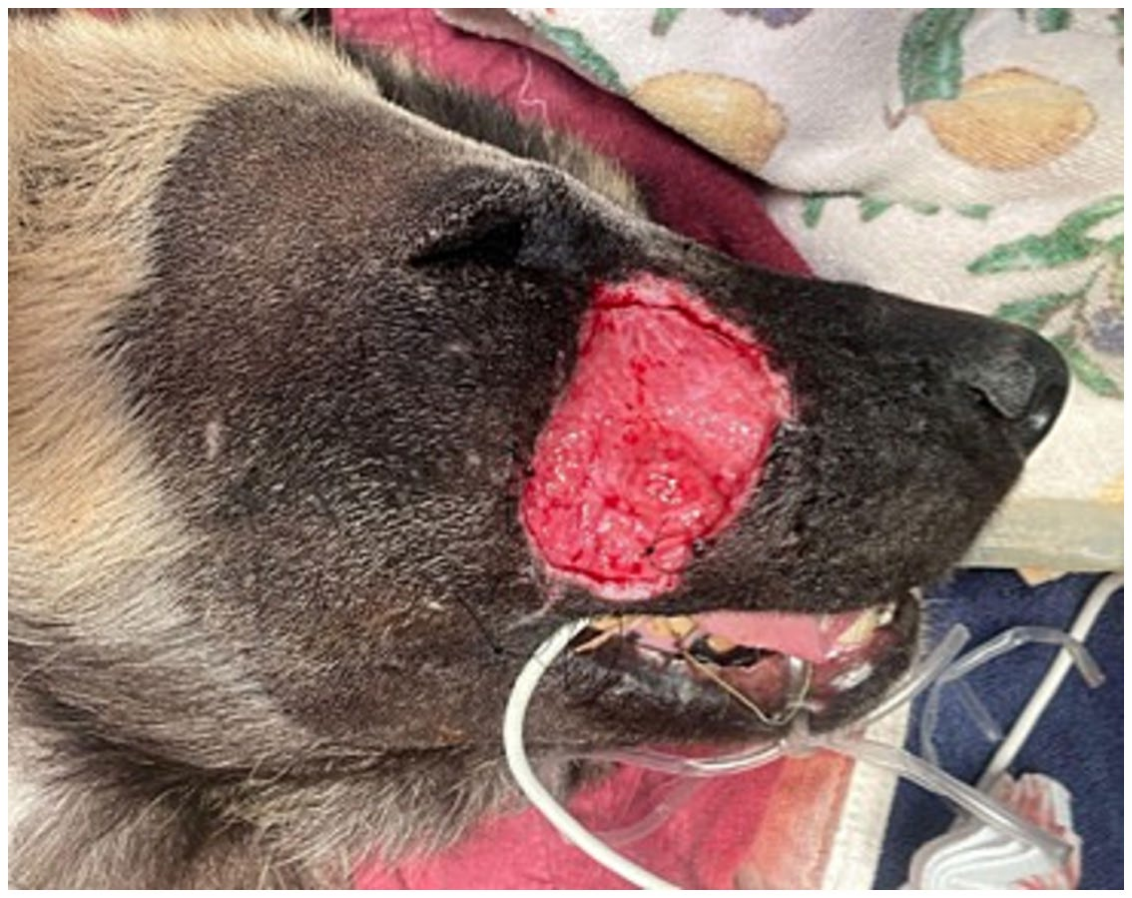
Five days following open wound management with tie-over bandage and routine bandage changes every 2 days with limited exudative discharge and healthy granulation bed.

**Figure 3:**
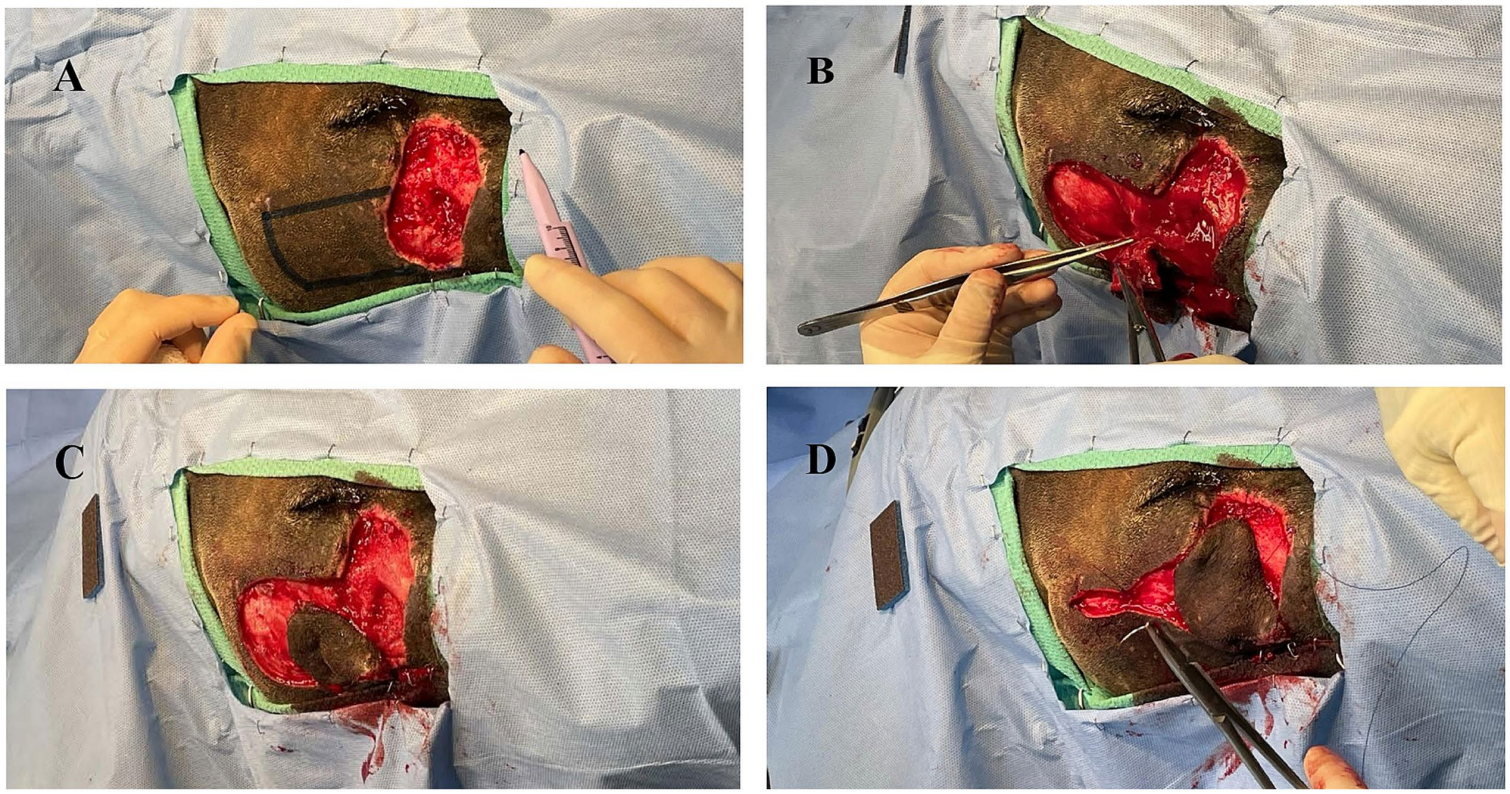
**(A)** Initial surgical field with planned donor flap margins digitally enhanced. **(B)** Elevation of donor tissue for transpositional skin flap. **(C)** Donor tissue shrinkage following creation of transpositional skin flap. **(D)** Rotation of donor tissue into the recipient bed with partial closure of the donor defect.

**Figure 4:**
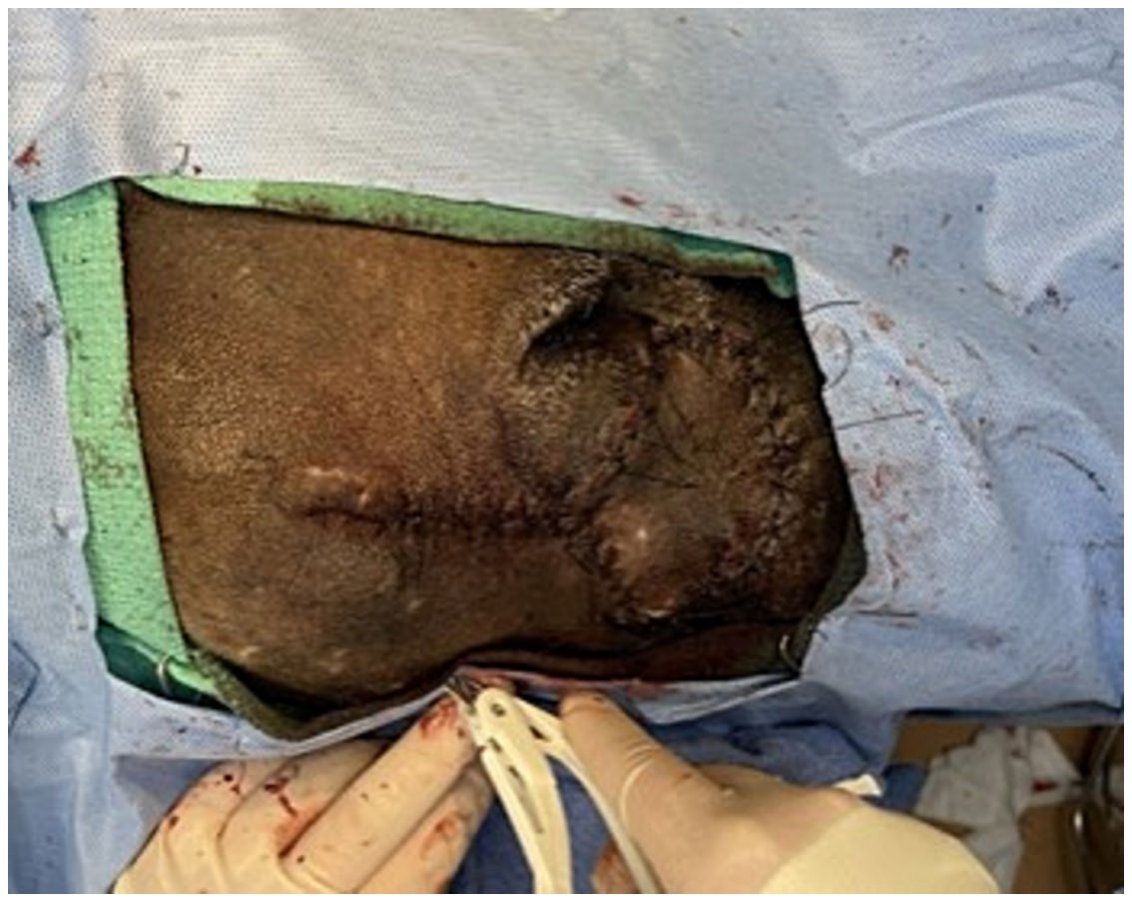
Immediate post-operative closure of facial wound using a transpositional skin flap.

After 14 days from initial clinical signs (facial swelling), the AWD was anesthetized using the aforementioned protocol for evaluation and surgical closure of the facial defect. The historic facial swelling, hyperthermia, and leukocytosis had resolved, and a healthy granulation tissue bed had formed (Figure 2). Perioperative antibiotics cefazolin (24 mg/kg IV once; West-ward) and intravenous fluids (Lactated Ringer’s Solution 5 mL/kg/h; Nova-Tech) were administered. A local subdermal plexus transpositional flap (2.5 cm wide and 4.5 cm long) was created. The flap base extended from the most ventral portion of the defect to half the distance in a dorsoventral direction along the wound. The edges of the flap were created parallel to these points, extending from the wound towards the caudal face, parallel to the zygomatic arch. The recipient bed wound edges were sharply transected, and the flap was rotated approximately 90 degrees onto the granulation bed (Figures 3A–D). The subcutaneous tissue was apposed with 2–0 and 3–0 PDS (polydiaxonone violet monofilament ethalloy; Eticon) in a simple interrupted pattern and the skin was apposed using 3-0 nylon in a simple interrupted and cruciate suture pattern (Figure 4). Liposomal bupivacaine (Nocita, 0.83 mg/kg SQ once; Elanco) was administered during closure within the subcutaneous layer. Postoperative medications included buprenorphine (Simbadol; 0.7 mg/kg SQ once; Zoetis), meloxicam (0.1 mg/kg SQ once), and ceftiofur crystalline free acid (Excede; 10 mg/kg SQ once; Zoetis). The AWD recovered from anesthesia uneventfully at the zoo’s hospital and was administered trazodone (3 mg/kg PO twice daily for 2 weeks; Teva Pharmaceuticals) to reduce unwanted activity and stress and thus minimize potential self-induced trauma to the flap.

**Figure 5:**
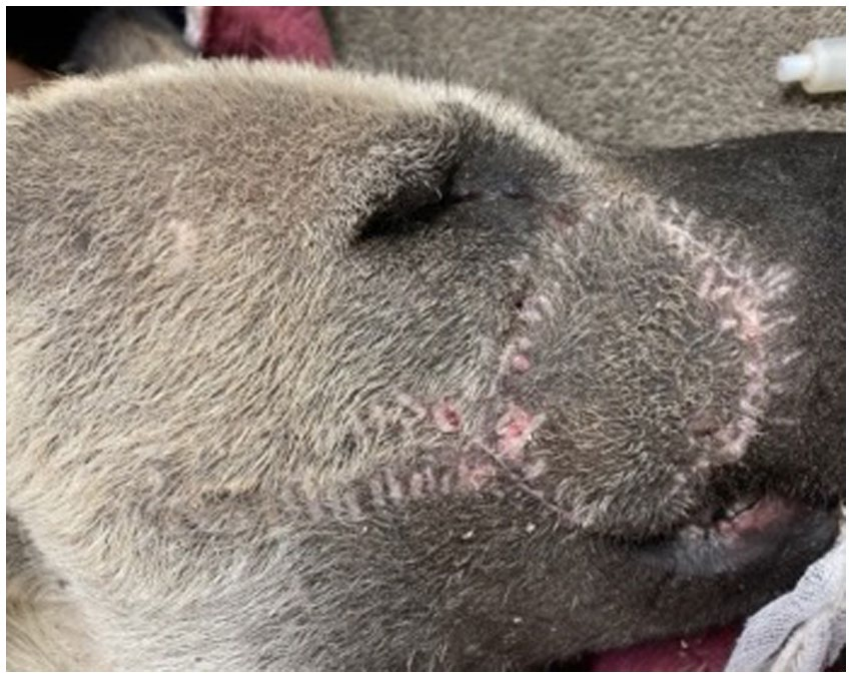
Transpositional skin flap healing at 17 days post-operatively resulting in good cosmesis and healing. All areas appear well apposed with no overt changes in previous cosmesis or functional dehiscence. Focal area of flap necrosis was appreciated rostral to the medial canthus of the right eye (<5 mm).

Postoperatively, the patient was visually monitored with no reported concerns. A minimum of 2 weeks of healing time was deemed appropriate to reduce risk to the surgical flap if any conspecific aggression occurred during pack reintroductions. Seventeen days postoperatively, the AWD was anesthetized using the previously described protocol for a final examination, suture removal, and to facilitate transport of the AWD back to the animal’s enclosure with its pack. Skin sutures were removed, and a 5 mm region of incomplete healing was appreciated, but the remainder of the flap and wound had completely healed (Figure 5). The dog was administered meloxicam (0.1 mg/kg SQ once), naltrexone (0.2 mg/kg IM; ZooPharm), atipamezole (0.06 mg/kg IM), and flumazenil (0.01 mg/kg IV; West-Ward) and recovered from anesthesia without complication. The AWD was reintroduced to the pack within an hour following anesthetic recovery and immediately returned to its previously held position in the social hierarchy with no adverse conspecific interactions noted.

## Discussion

This case report describes the management and successful surgical repair of a large facial wound defect in an AWD using a transpositional skin flap technique. The goals of this case report are to highlight that surgical intervention allowed for healing with few anesthetic events, reduced patient interactions and rapid healing time, and successful reintroduction to the social group. Various other surgical corrections could have been selected, but a transposition skin flap was selected as the means of closure for three main reasons. Firstly, transpositional skin flaps have a reported high success rate and low dehiscence rate when utilized in domestic dogs (5, 6, 8). We anticipated that despite species differences, we would appreciate a similar outcome when this technique was applied to the AWD. A second factor contributing to the surgical technique decision was the goal to achieve complete tissue healing in the shortest amount of time and with the fewest anesthetic events possible to reduce both stress to the animal and time to reintroduction to the pack. Complete healing with surgical closure was accomplished in 17 days in this case; by comparison, healing by second intention of a wound of this size would have theoretically taken a much longer amount of time. In domestic dogs, re-epithelialization has been cited as occurring at a rate of approximately 1 mm/day and this rate theoretically could be applied to an AWD to determine an approximate healing time (10). Thirdly, we aimed to maintain cosmesis due to the hypothesized importance of visual individual recognition in AWDs, which in turn may help preserve social structure within the pack (1).

Initially, the defect was managed as an open wound with tie-over bandages. This therapy was pursued to allow time for declaration of devitalized tissue, resolution of tissue edema, and development of granulation tissue. In addition, this time allowed for assessment of pending diagnostics, including bacterial cultures and histopathologic evaluation. Prior to performing a flap technique of any kind, the recipient defect should be free of bacterial colonization and devitalized tissue. Challenges of open wound management for an AWD are not dissimilar from domestic dogs except for the requirement of general anesthesia for bandage changes that may be accomplished awake or with light sedation in domestic animals, depending on individual temperament.

This report demonstrates the benefits that surgical skin flap procedures can provide to zoological species with extensive wounds. The skin flap in this case resulted in good cosmesis and rapid successful reintegration of the AWD back into the social group, without negative hierarchical shifts and conspecific aggression.

## Conclusion

This case report describes the use of a local subdermal transposition skin flap in a novel species to aid in closure of a large facial defect. This procedure resulted in an excellent outcome and swift return of the patient to its social pack.

## Data Availability

The raw data supporting the conclusions of this article will be made available by the authors, without undue reservation.
